# Static and dynamic balance of children and adolescents with sensorineural hearing loss

**DOI:** 10.1590/S1679-45082017AO3976

**Published:** 2017

**Authors:** Renato de Souza Melo, Sônia Elvira dos Santos Marinho, Maryelly Evelly Araújo Freire, Robson Arruda Souza, Hélio Anderson Melo Damasceno, Maria Cristina Falcão Raposo

**Affiliations:** 1 Universidade Federal de Pernambuco, Recife, PE, Brazil.; 2 Hospital Mestre Vitalino, Caruaru, PE, Brazil.; 3 Instituto Aggeu Magalhães, Fundação Oswaldo Cruz, Recife, PE, Brazil.; 4 Faculdade de Ciências Médicas, Campina Grande, PB, Brazil.; 5 Centro de Promoção à Saúde, UNIFISIO, Caruaru, PE, Brazil.

**Keywords:** Child behavior, Psychomotor performance, Motor skills, Ear, inner, Deafness, Comportamento infantil, Desempenho psicomotor, Destreza motora, Orelha interna, Surdez

## Abstract

**Objective:**

To assess the static and dynamic balance performance of students with normal hearing and with sensorineural hearing loss.

**Methods:**

A cross-sectional study assessing 96 students, 48 with normal hearing and 48 with sensorineural hearing loss of both sexes, aged 7 and 18 years. To evaluate static balance, Romberg, Romberg-Barré and Fournier tests were used; and for the dynamic balance, we applied the Unterberger test.

**Results:**

Hearing loss students showed more changes in static and dynamic balance as compared to normal hearing, in all tests used (p<0.001). The same difference was found when subjects were grouped by sex. For females, Romberg, Romberg-Barré, Fournier and Unterberger test p values were, respectively, p=0.004, p<0.001, p<0.001 and p=0.023; for males, the p values were p=0.009, p<0.001, p<0.001 and p=0.002, respectively. The same difference was observed when students were classified by age. For 7 to 10 years old students, the p values for Romberg, Romberg-Barré and Fournier tests were, respectively, p=0.007, p<0.001 and p=0.001; for those aged 11 and 14 years, the p values for Romberg, Romberg-Barré, Fournier and Unterberger tests were p=0.002, p<0.001, p<0.001 and p=0.015, respectively; and for those aged 15 and 18 years, the p values for Romberg-Barré, Fournier and Unterberger tests were, respectively, p=0.037, p<0.001 and p=0.037.

**Conclusion:**

Hearing-loss students showed more changes in static and dynamic balance comparing to normal hearing of same sex and age groups.

## RESUMO

**Objetivo:**

Avaliar o desempenho do equilíbrio estático e dinâmico de escolares ouvintes e com perda auditiva sensório-neural.

**Métodos:**

Estudo de corte transversal que avaliou 96 escolares, sendo 48 ouvintes e 48 com perda auditiva sensório-neural, de ambos os sexos, na faixa etária entre 7 e 18 anos. Para a avaliação do equilíbrio estático, foram utilizados os testes de Romberg, Romberg-Barré e Fournier, e, para avaliar o equilíbrio dinâmico, foi usado o teste de Unterberger.

**Resultados:**

Os escolares com perda auditiva apresentaram maior ocorrência de alterações no equilíbrio estático e dinâmico, comparados aos ouvintes, em todos os testes empregados (p<0,001). O mesmo foi observado quando os escolares foram agrupados pelos sexos. Para o sexo feminino, os valores de p para os testes de Romberg, Romberg-Barré, Fournier e Unterberger foram, respectivamente, p=0,004, p<0,001, p<0,001 e p=0,023; para o sexo masculino, foram p=0,009, p<0,001, p<0,001 e p=0,002. A mesma diferença foi observada quando os escolares foram estratificados pelas faixas etárias. Para aqueles de 7 a 10 anos, os valores de p foram, respectivamente, para os testes de Romberg, Romberg-Barré e Fournier p=0,007, p<0,001 e p=0,001; entre 11 e 14 anos, para os testes de Romberg, Romberg-Barré, Fournier e Unterberger, foram, respectivamente p=0,002, p<0,001, p<0,001, p=0,015; entre 15 e 18 anos, para os testes Romberg-Barré, Fournier e Unterberger foram, respectivamente, p=0,037, p<0,001 e p=0,037.

**Conclusão:**

Os escolares com perda auditiva apresentaram maior ocorrência de alterações no desempenho do equilíbrio estático e dinâmico que os ouvintes, do mesmo sexo e faixa etária.

## INTRODUCTION

Body balance consists of maintaining the center of gravity within the base of support defined by the feet, and it may be static or dynamic.^(^ ^1^ ^)^ In static balance, the base of support remains fixed while the center of gravity moves. In this case, the sense of balance maintains the center of gravity within the base of support defined by the feet. Whereas, in dynamic balance, both the center of gravity and the base of support are in constant motion, and the center of gravity never aligns itself to the base of support during the stance phase of the movement.^(^ ^2^ ^)^

To maintain human body balance, anatomical and functional integrity of the vestibular system, located in the inner ear, is essential.^(^ ^3^ ^)^ The vestibulo-cochlear system has a dual function — the cochlea is responsible for the auditory function, and the vestibular system is responsible for body balance.^(^ ^4^ ^)^ However, hearing ability is actually a secondary feature, because the primary function of the auditory organ is to maintain body balance.^(^ ^5^ ^)^

However, when the vestibular system presents some type of disorder, body balance can be affected, as in some individuals with sensorineural hearing loss.^(^ ^6^ ^)^ As the vestibular system and the cochlea are anatomically very close to each other and may be vulnerable to the same harmful agents, children with internal ear injuries may present vestibular problems concomitant with sensorineural hearing loss.^(^ ^7^ ^-^ ^9^ ^)^ Furthermore, several studies indicate that vestibular dysfunction is a frequent finding in the otoneurological evaluation of children with sensorineural hearing loss.^(^ ^10^ ^,^ ^11^ ^)^

Vestibular disorders in childhood are not as rare as believed and may affect the acquisition of neuromotor skills, or interfere mainly in the sensory integration of the vestibular system. Children with vestibular disorders may report imbalance, dizziness, gait disorders and present with other manifestations, such as falls, dizziness, vertigo and frequent bumping into people/objects, which hinder the acquisition of motor skills typical of their age, such as riding a bicycle, skipping rope, playing “hopscotch” or even using playground toys.^(^ ^12^ ^)^

In view of the above, children with sensorineural hearing loss appear to present alterations in sensory information provided by the vestibular system, possibly due to internal ear injury.^(^ ^13^ ^)^ This condition may favor body balance changes in this population. In spite of these evidences, there are scarce data in the literature on balance in children with sensorineural hearing loss compared to normal-hearing children, particularly in relation to reporting the dynamic balance performance, which justifies the present study.

## OBJECTIVE

To evaluate static and dynamic balance performance in normal-hearing students compared to children with sensorineural hearing loss.

## METHODS

This is a cross-sectional study, in which a previous survey was carried out with the manager of a school dedicated to the education of children with special needs to delimit determine the sample size, to find out how many students with sensorineural hearing loss were enrolled in the age range covered by the study, and which of them fit the eligibility criteria. Normal hearing students were recruited from another educational institution. Both schools belonged to the state education network, had similar socioeconomic profiles, and were located in the city of Caruaru (PE).

There was a predominance of female students and some age groups, which made it difficult to enlarge the study sample. Matching was possible for 48 students, by sex and age group. Thus, 96 students participated in this study, of which 48 in the Normal Hearing Group (NHG) and 48 in the Sensorineural Hearing Loss Group (SHLG), of both sexes, aged between 7 and 18 years.

The students were recruited by sequential convenience sampling, matched by sex and age group, and selected by draws made by their teachers, who were not aware of the characteristics of this study. The draws took place in the classroom, in front of all the students and researchers of the study.

The NHG was composed of 48 students, 24 male and 24 female, with two representatives of both sexes for each age group. The exclusion criteria adopted for this group were presence of neurological, physical, visual or mental deficiency, hearing complaint, and leg length discrepancy greater than 2cm, by actual measurement and apparent measurement of lower limbs.

The SHLG comprised 48 students with sensorineural hearing loss, 24 male and 24 female, with two representatives of both sexes in each age group. The exclusion criteria for this group were presence of neurological, physical, visual or mental deficiency, and leg length discrepancy greater than 2cm, by actual measurement and apparent measurement of lower limbs conducted previously by the evaluators in each group. Other inclusion criteria for the SHLG were having received a clinical diagnosis of sensorineural hearing loss, issued by means of a medical report, and mastering the Brazilian Language of Signals (LIBRAS). This last criterion was employed to ensure that the commands related to the demands of the method would really be understood by all students with hearing loss. All students had access to learning LIBRAS in their school.

The inclusion criteria for both groups were: to be regularly enrolled in one of the schools collaborating with the research and to be in the age group between 7 and 18 years.

The information for the eligibility criteria of this study was obtained from the parents of the students, in interviews with the researchers; from the students’ school records, furnished by the school manager; and in the physical evaluation performed prior to the balance performance assessment.

The procedures that preceded the static and dynamic balance evaluation were previously explained and demonstrated by the researchers to the normal hearing students, orally, and to the students with hearing loss, using LIBRAS, by one of the researchers who is an interpreter of this language.

In both groups, the students were evaluated one by one by the same physical therapist in a reserved room of the school, using four clinical tests to detect changes in static and dynamic balance. To evaluate static balance, the Romberg, Romberg-Barré and Fournier tests were used, and the Unterberger test was used to evaluate dynamic balance.

### Static balance evaluation

In the Romberg test,^(^ ^14^ ^)^ the volunteer remained standing up, barefoot, with the feet together in parallel position, the arms along the body, with the eyes closed, for one minute.

The Romberg-Barré test^(^ ^14^ ^)^ was performed similarly to the Romberg test, but with one foot directly in front of the other on the ground, in a straight line.

A loss of balance and the inability to maintain the initial position of the test characterized a change in static balance for Romberg test and Romberg-Barré test.

In the Fournier test,^(^ ^14^ ^)^ the volunteer remained standing up, barefoot, with unipodal support, standing up on the dominant leg, with the eyes closed, for 30 seconds. According to the test, those who could not remain in unipodal support during the established time were classified as having a change in static balance.

### Dynamic balance evaluation

In the Unterberger test,^(^ ^14^ ^)^ the students marched in place with their eyes closed, raising their knees up to approximately 45° and their hips up to 90°, performing 50 steps (one per second) with their arms extended in front of them at 90°. The volunteers who showed a right or left rotation equal or superior to 45° were classified as having a change in dynamic balance.

Static and dynamic balance data were recorded in a standardized card, containing the student’s identification, age, grade, school, lateral dominance and performance in all tests.

Data were analyzed by Pearson’s χ^2^ test or Fisher’s exact test. The statistical significance level was set at p<0.05, and the Statistical Package for the Social Sciences (SPSS), version 18.0, was used to perform the statistical analysis of these data.

This study was evaluated and approved by the Research Ethics Committee of the *Hospital da Restauração*, according to the final protocol number CAAE: 1700.0.000.102-11, as provided by Resolution 466/12 of the National Health Council.

## RESULTS

The present study evaluated static and dynamic balance in 48 normal hearing students and 48 students with sensorineural hearing loss, with mean age of 12.5±3.5 years in each group, as shown in table 1.

**Table 1:** Sample characteristics

Characteristics	Normal hearing	Hearing loss	p value
	n (%)	n (%)	
Volunteers	48 (100)	48 (100)	-
Sex			
Female	24 (50)	24 (50)	-
Male	24 (50)	24 (50)	-
Age (years)	12.5±3.5 (100)	12.5±3.5 (100)	-
Dominant side			
Right-handed	45 (93.7)	41 (85.4)	0.181^*^
Left-handed	3 (6.3)	7 (14.6)	
Hearing loss severity			
Mild and moderate	-	4 (8.3)	-
Severe	-	44 (91.7)	-

^*^ Pearson χ^2^ test.

Students with hearing loss showed greater occurrence of static and dynamic balance alterations than normal hearing children, with statistical differences in all the tests used, as shown in table 2.

**Table 2:** Changes in static and dynamic balance performance of normal-hearing and sensorineural hearing loss students

Test	Normal hearing (n=48)	Hearing loss (n=48)	p value	Prevalence rate	95%CI
Romberg	0 (0)	15 (31.3)	<0.001^*^	-	-
Romberg-Barré	2 (4.2)	37 (77.1)	<0.001^†^	18.5	4.72-72.4
Fournier	9 (18.8)	42 (87.5)	<0.001^†^	4.67	2.56-8.49
Unterberg	2 (4.2)	19 (39.6)	<0.001^*^	9.50	2.34-38.5

* Fisher’s exact test; ^†^ Pearson χ^2^ test.

95%CI: 95% confidence interval.

The same difference was observed when the students were grouped by sex (Table 3). The same was observed when the students were stratified by their age group. In the evaluation of static balance, the data showed differences in all age groups in the Romberg-Barré and Fournier tests; in the evaluation using the Romberg test, differences were observed in the age groups from 7 to 14 years. The dynamic balance evaluation showed differences among the students evaluated, in the age groups from 11 to 18 years, as shown in table 4.

**Table 3:** Changes in static and dynamic balance performance of normal-hearing and sensorineural hearing loss students, per sex

Test	Female (n=48)			Male (n=48)		
	
	Normal hearing (n=24) n (%)	Hearing loss (n=24) n (%)	p value	Normal hearing (n=24) n (%)	Hearing loss (n=24) n (%)	p value
Romberg	0 (0)	8 (33.6)	0.004^*^	0 (0)	7 (29.4)	0.009^*^
Romberg-Barré	1 (4.2)	16 (67.2)	<0.001^†^	1 (4.2)	21 (87.6)	<0.001^*^
Fournier	5 (20.8)	22 (91.8)	<0.001^†^	4 (16.7)	20 (83.4)	<0.001^*^
Unterberger	1 (4.2)	8 (33.6)	0.023^*^	1 (4.2)	11 (45.8)	0.002^*^

* Fisher’s exact test; ^†^ Pearson χ^2^ test.

**Table 4:** Changes in static and dynamic balance performance of normal-hearing and sensorineural hearing loss students, per age group

Test	7-10 years (n=32)			11-14 years (n=32)			15-18 years (n=32)		
		
	Normal hearing (n=16)	Hearing loss (n=16)	p value	Normal hearing (n=16)	Hearing loss (n=16)	p value	Normal hearing (n=16)	Hearing loss (n=16)	p value
Romberg	0 (0%)	7 (43.8)	0.007^*^	0 (0)	8 (50)	0.002^*^	0 (0)	0 (0)	--
Romberg-Barré	0 (0%)	16 (100)	<0.001^†^	1 (6.3)	14 (87.6)	<0.001^†^	1 (6.3)	7 (43.8)	0.037^*^
Fournier	5 (31.3%)	14 (87.6)	0.001^†^	3 (18.8)	14 (87.6)	<0.001^†^	1 (6.3)	14 (87.6)	<0.001^†^
Unterberger	0 (0)	4 (25)	0.101^*^	1 (6.3)	8 (50)	0.015^*^	1 (6.3)	7 (43.8)	0.037^*^

* Fisher’s exact test; ^†^ Pearson χ^2^ test.

## DISCUSSION

In the present study, we observed differences between static and dynamic balances in the students evaluated, and the SHLG showed greater occurrence of alterations in this motor skill than the NHG.

Likewise, Jafari et al.,^(^ ^15^ ^)^ evaluated static balance in 60 children, 30 of whom were normal hearing children and 30 were children with sensorineural hearing loss, in the age group of 6 to 9 years, using six balance tests of the Bruininks-Oseretsky Test of Motor Proficiency, and found differences in static balance performance among the groups. Children with hearing loss also had poorer balance than normal hearing children.

An et al.,^(^ ^16^ ^)^ analyzed static balance in 114 students, of which 57 were normal hearing children and 57 were children with sensorineural hearing loss, aged 4 to 14 years, using the unipodal support test. They concluded that children with hearing loss presented more static balance instability than normal hearing children, as we also observed in this study.

In addition to the changes observed in the SHLG, related to static balance, in this study we also observed changes in dynamic balance performance. The SHLG presented more changes in dynamic balance than normal hearing children, taking into consideration the variables sex and age group 11 and 18 years.

Gayle et al.,^(^ ^17^ ^)^ evaluated dynamic balance in 40 children, of which 20 were normal hearing children and 20 were children with sensorineural hearing loss, of both sexes and with a mean age of 12.3±5.6 years. In their study, children with hearing loss showed lower dynamic balance performance than normal hearing children, a fact also observed by the present study.

The scarcity of data on dynamic balance performance of children with sensorineural hearing loss made it difficult to discuss this finding, but results from other studies reinforce the findings of the present study, regarding dynamic balance in children and adolescents with sensorineural hearing loss.

Melo et al.,^(^ ^18^ ^)^ compared body balance and gait in students with hearing loss of both sexes, between 7 and 17 years. The authors did not find body balance differences in the evaluated students, unlike the findings of this study. However, differences were observed in gait performance, whose tests were conducted in dynamic situations. The authors believe that children with hearing loss cannot create posture strategies to circumvent the test when performed in dynamic situations, which could justify the findings of this study concerning changes in dynamic balance.

Likewise, Atasavun Uysal et al.,^(^ ^19^ ^)^ evaluated the gait of 20 normal hearing children with mean age of 12.2±2.5 years and 20 children with hearing loss and mean age of 9.25±0.9 years. The gait evaluation was done by spraying the children’s feet and instructing them to walk across a dark platform in a straight line. By demarcating the children’s steps on the platform, the authors evaluated the length and width of the steps, the angle of the feet, the number of steps per minute, and the speed of the gait. In all these outcomes, children with hearing loss showed lower performance than normal hearing children, agreeing with the findings of this study.

The changes in static and dynamic balance found in students with hearing loss in this study may, if not treated, negatively influence the performance of motor skills that depend on an adequate body balance to be performed satisfactorily, such as walking, running and jumping, and this may interfere with the physical fitness and recreational and/or sports practices of these children.

In this context, Hartman et al.,^(^ ^20^ ^)^ evaluated the sports participation of 42 children with hearing loss, and reported that they had greater limitation in manual dexterity (62%), ball skills (52%), and balance skills (45%). The authors believed that improving these motor skills, early in childhood, could contribute in a positive way in the school context and in the sports and social practices of these children.

In addition to the data obtained in the groups alone, some variables were also analyzed in this study, and one of the most relevant findings was observed in the variable age group. An extensive age group was employed to assess body balance performance in adolescence, given the scarce data in the literature. There was an improvement in body balance, in view of a lower frequency of body balance changes in older age groups, although differences among groups remained. This suggests that interventions in this population should be conducted not only in childhood but also in adolescence, and this may be incorporated into the school environment.

The groups presented differences for both sexes. However, boys presented more changes in body balance than girls, corroborating the findings of Dorneles et al.,^(^ ^21^ ^)^ who evaluated and compared body balance in healthy adolescents according to sex and observed that females had better body balance stability than males. In addition, the girls in this study were more focused on the tests than the boys, who showed more anxiety while performing the tests, which may justify the findings related to the variable sex in this study.

Considering the above, this study identified static and dynamic balance changes in students with sensorineural hearing loss compared to normal hearing children of the same sex and age group. We suggest that such changes may occur due to an inadequate sensory organization in these children, due to the possible involvement of the vestibular system, caused by inner ear injury.

Supporting this hypothesis, Guilder et al.,^(^ ^22^ ^)^ reported that children with severe and profound hearing loss often had vestibular system hypoactivity. Similarly, Lavinsky^(^ ^23^ ^)^ described children with profound hearing loss and high prevalence of vestibular dysfunction in their study. These data could justify the findings of this study, considering that 91.7% of children with hearing loss in this study had severe and profound hearing loss. Other researches also reported that vestibular dysfunction is a frequent finding in otoneurological evaluations of children with sensorineural hearing loss.^(^ ^24^ ^-^ ^26^ ^)^

It is worth noting that the evaluation of the vestibular function was not performed in the hearing-loss students in this study, because a computerized vectoelectronystagmography is a high cost exam and is not available in the public health system of the city where the study was carried out, besides the fact that this project had no funding. This was, therefore, a limitation for any conclusions on the vestibular involvement in children and adolescents with hearing loss in this study.

However, it is believed that every child with a clinical diagnosis of sensorineural hearing loss should undergo vestibular testing regardless of age and sex, and even in the absence of dizziness or vertigo.^(^ ^27^ ^)^

Given the importance of examining the vestibular function, we suggest that future studies make this association between static and dynamic balance performance in children with hearing loss, with and without associated vestibular dysfunction, in order to identify the real influence of each of these systems (auditory and vestibular) in the balance performance of this population.

One of the major contributions of this study was to provide data on dynamic balance performance of children and, especially, adolescents with hearing loss compared to normal hearing children, given the scarce data in the literature. In addition, it presented a homogeneous sample, which was pair-matched and much more representative than the study samples found in the literature.

According to the findings of this study, students with sensorineural hearing loss presented more static and dynamic balance alterations than normal hearing students of the same sex and age group.

This may be related to the involvement of the vestibular system due to inner ear injury. These changes may arise since early childhood, stressing the importance of raising awareness among health professionals and, above all, among hearing health and neuromotor development specialists, such as otolaryngologists (ENT specialists), audiologists and speech therapists, and pediatricians, so that these professionals have a more general approach to children with hearing loss and exchange information, focusing not only on the hearing aspect, but also on the children’s neuromotor development and performance, in view of the observed alterations, which are often unknown by many of these professionals.

Therefore, we stress the need of preventive programs in students health that reinforce the practice of specific physical exercises, with neuromotor evaluations of body balance and motor skills, in a periodic way, as well as early specific interventions, if necessary.

All these tasks are physical therapy responsibilities, reflecting the importance of the physicalotherapist in the school environment. These interventions could be incorporated into the school daily routine, in institutions that serve this population and in multiprofessional teams, seeking to adapt and/or improve body balance, motor performance and quality of life of children and adolescents with sensorineural hearing loss.

## CONCLUSION

Students with sensorineural hearing loss had a higher occurrence of changes in static and dynamic balance than normal hearing students of the same sex and age group.
